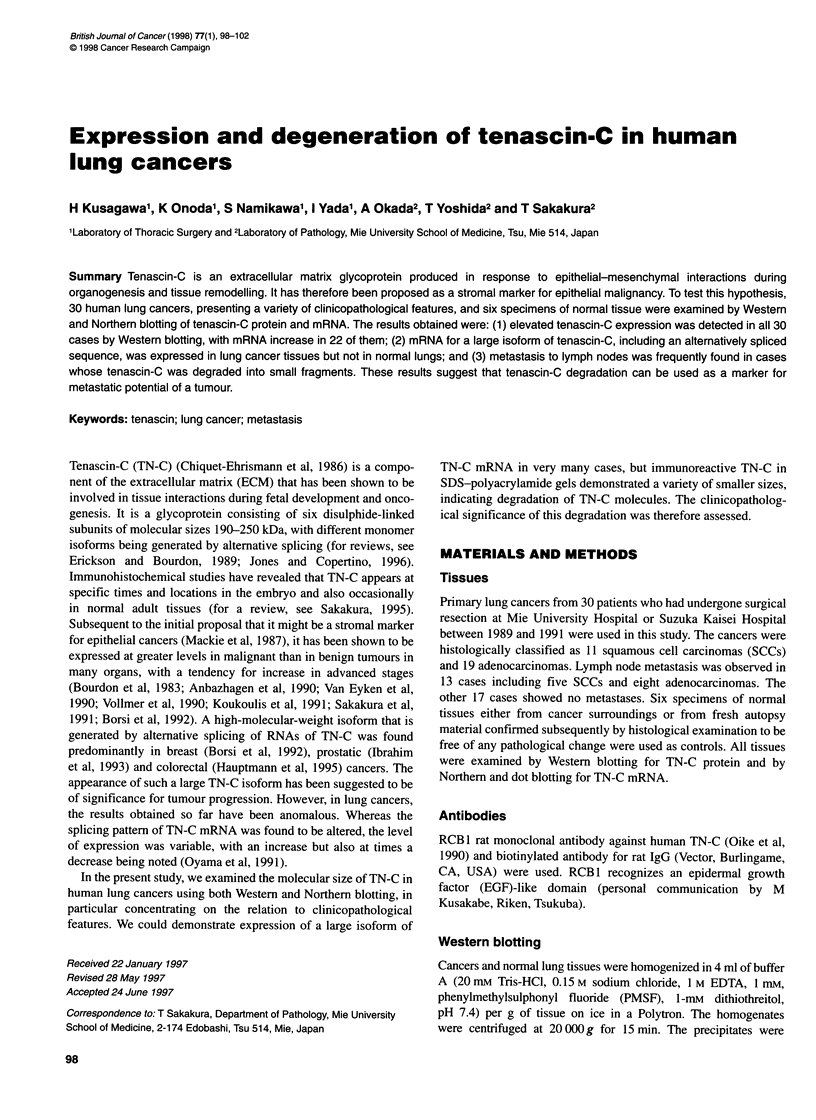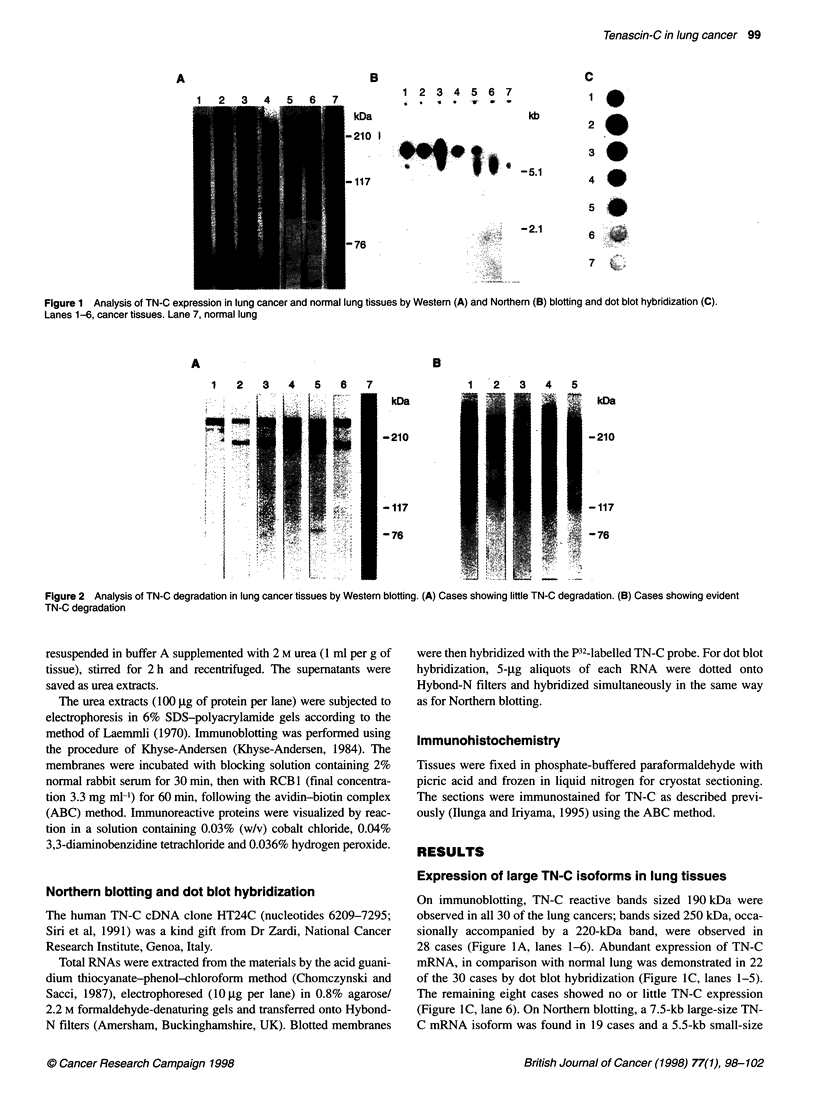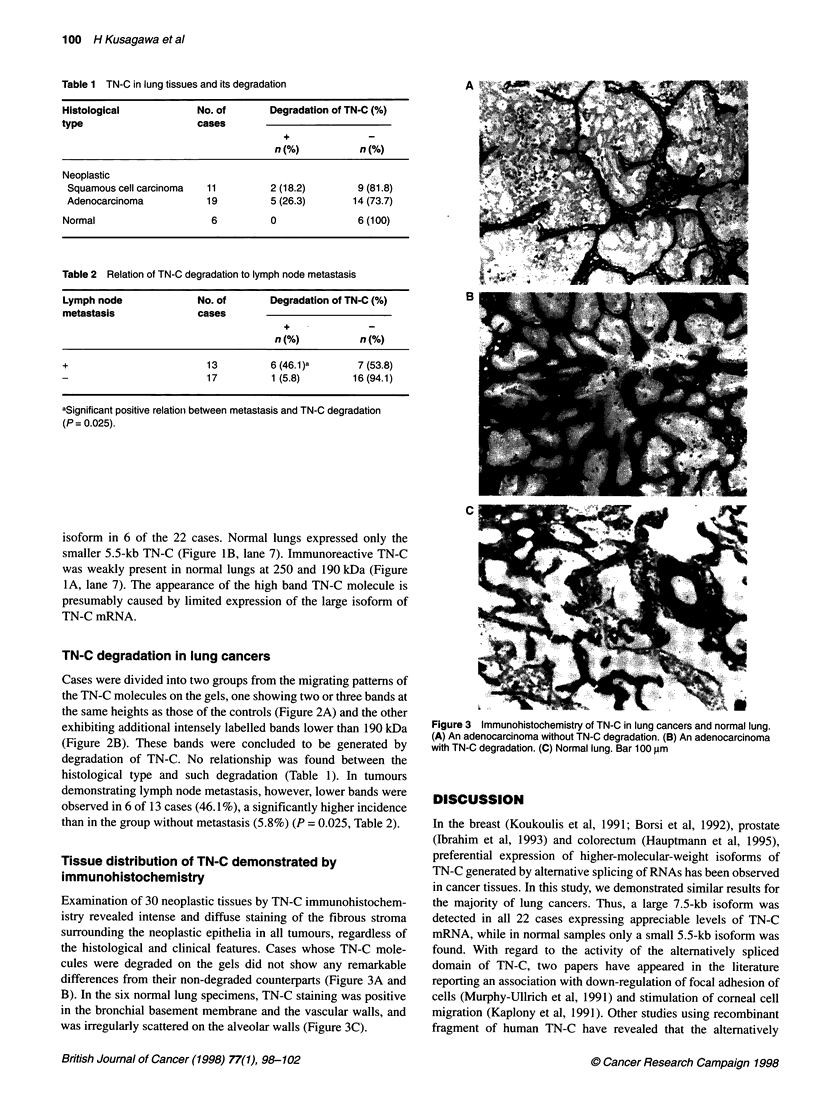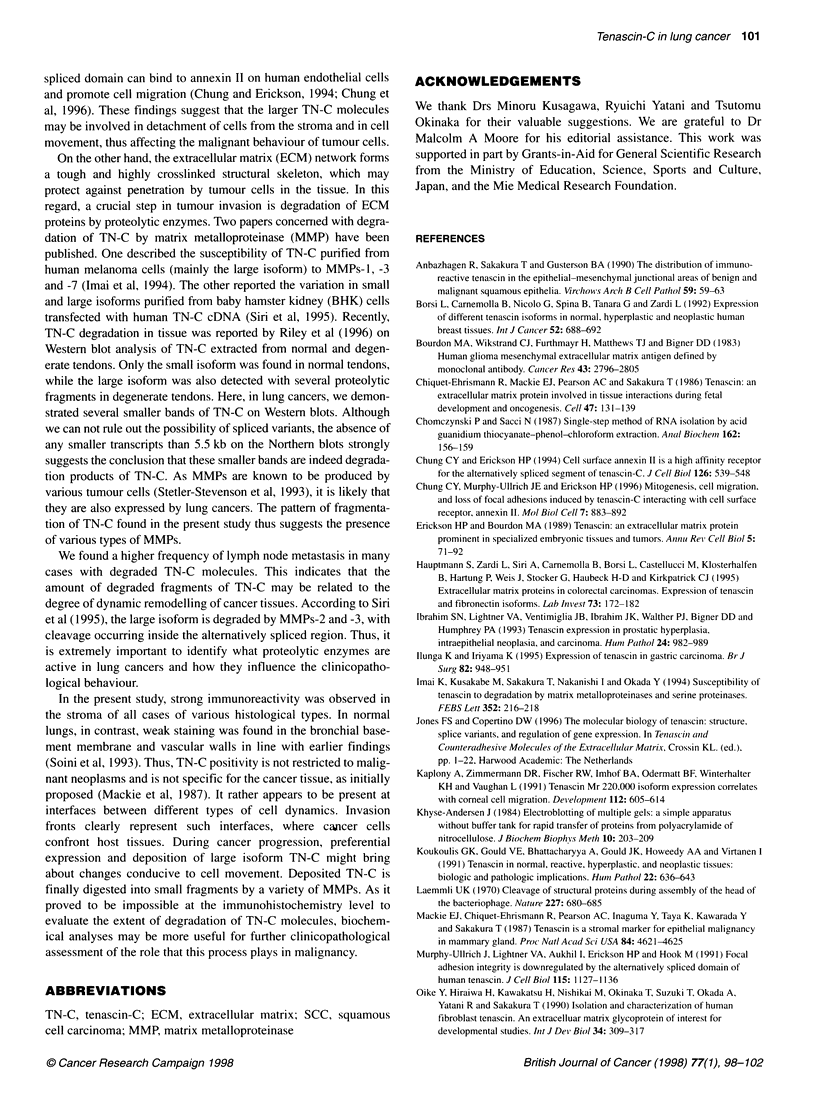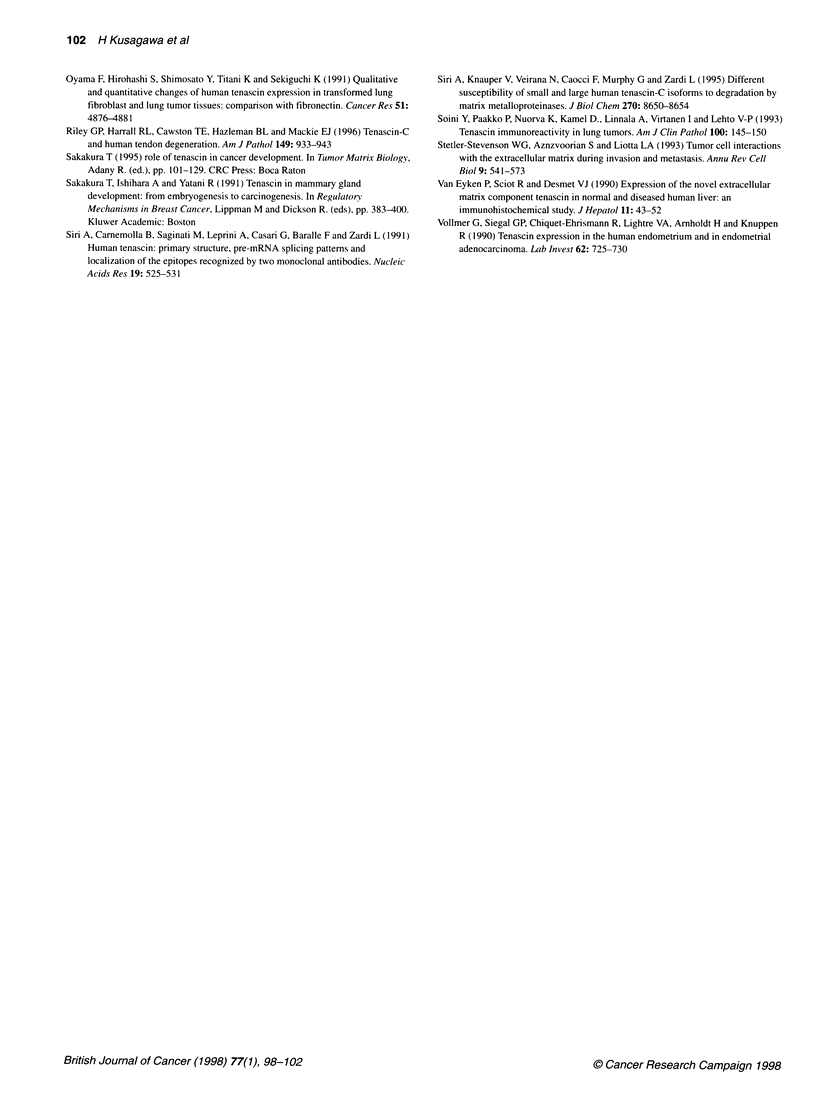# Expression and degeneration of tenascin-C in human lung cancers.

**DOI:** 10.1038/bjc.1998.15

**Published:** 1998

**Authors:** H. Kusagawa, K. Onoda, S. Namikawa, I. Yada, A. Okada, T. Yoshida, T. Sakakura

**Affiliations:** Laboratory of Thoracic Surgery, Mie University School of Medicine, Tsu, Japan.

## Abstract

**Images:**


British Journal of Cancer (1998) 77(1), 98–102
© 1998 Cancer Research Campaign

### Expression and degeneration of tenascin-C in human lung cancers

**H Kusagawa[1], K Onoda[1], S Namikawa[1], I Yada[1], A Okada[2], T Yoshida[2] and T Sakakura[2]**

[1]Laboratory of Thoracic Surgery and [2]Laboratory of Pathology, Mie University School of Medicine, Tsu, Mie 514, Japan

**Summary** Tenascin-C is an extracellular matrix glycoprotein produced in response to epithelial–mesenchymal interactions during organogenesis and tissue remodelling. It has therefore been proposed as a stromal marker for epithelial malignancy. To test this hypothesis, 30 human lung cancers, presenting a variety of clinicopathological features, and six specimens of normal tissue were examined by Western and Northern blotting of tenascin-C protein and mRNA. The results obtained were: (1) elevated tenascin-C expression was detected in all 30 cases by Western blotting, with mRNA increase in 22 of them; (2) mRNA for a large isoform of tenascin-C, including an alternatively spliced sequence, was expressed in lung cancer tissues but not in normal lungs; and (3) metastasis to lymph nodes was frequently found in cases whose tenascin-C was degraded into small fragments. These results suggest that tenascin-C degradation can be used as a marker for metastatic potential of a tumour.

**Keywords:** tenascin; lung cancer; metastasis

Tenascin-C (TN-C) (Chiquet-Ehrismann et al, 1986) is a component of the extracellular matrix (ECM) that has been shown to be involved in tissue interactions during fetal development and oncogenesis. It is a glycoprotein consisting of six disulphide-linked subunits of molecular sizes 190–250 kDa, with different monomer isoforms being generated by alternative splicing (for reviews, see Erickson and Bourdon, 1989; Jones and Copertino, 1996). Immunohistochemical studies have revealed that TN-C appears at specific times and locations in the embryo and also occasionally in normal adult tissues (for a review, see Sakakura, 1995). Subsequent to the initial proposal that it might be a stromal marker for epithelial cancers (Mackie et al, 1987), it has been shown to be expressed at greater levels in malignant than in benign tumours in many organs, with a tendency for increase in advanced stages (Bourdon et al, 1983; Anbazhagen et al, 1990; Van Eyken et al, 1990; Vollmer et al, 1990; Koukoulis et al, 1991; Sakakura et al, 1991; Borsi et al, 1992). A high-molecular-weight isoform that is generated by alternative splicing of RNAs of TN-C was found predominantly in breast (Borsi et al, 1992), prostatic (Ibrahim et al, 1993) and colorectal (Hauptmann et al, 1995) cancers. The appearance of such a large TN-C isoform has been suggested to be of significance for tumour progression. However, in lung cancers, the results obtained so far have been anomalous. Whereas the splicing pattern of TN-C mRNA was found to be altered, the level of expression was variable, with an increase but also at times a decrease being noted (Oyama et al, 1991).

In the present study, we examined the molecular size of TN-C in human lung cancers using both Western and Northern blotting, in particular concentrating on the relation to clinicopathological features. We could demonstrate expression of a large isoform of TN-C mRNA in very many cases, but immunoreactive TN-C in SDS–polyacrylamide gels demonstrated a variety of smaller sizes, indicating degradation of TN-C molecules. The clinicopathological significance of this degradation was therefore assessed.

*Received 22 January 1997*
*Revised 28 May 1997*
*Accepted 24 June 1997*

*Correspondence to:* T Sakakura, Department of Pathology, Mie University School of Medicine, 2-174 Edobashi, Tsu 514, Mie, Japan

### MATERIALS AND METHODS

### Tissues

Primary lung cancers from 30 patients who had undergone surgical resection at Mie University Hospital or Suzuka Kaisei Hospital between 1989 and 1991 were used in this study. The cancers were histologically classified as 11 squamous cell carcinomas (SCCs) and 19 adenocarcinomas. Lymph node metastasis was observed in 13 cases including five SCCs and eight adenocarcinomas. The other 17 cases showed no metastases. Six specimens of normal tissues either from cancer surroundings or from fresh autopsy material confirmed subsequently by histological examination to be free of any pathological change were used as controls. All tissues were examined by Western blotting for TN-C protein and by Northern and dot blotting for TN-C mRNA.

### Antibodies

RCB1 rat monoclonal antibody against human TN-C (Oike et al, 1990) and biotinylated antibody for rat IgG (Vector, Burlingame, CA, USA) were used. RCB1 recognizes an epidermal growth factor (EGF)-like domain (personal communication by M Kusakabe, Riken, Tsukuba).

### Western blotting

Cancers and normal lung tissues were homogenized in 4 ml of buffer A (20 mM Tris-HCl, 0.15 M sodium chloride, 1 M EDTA, 1 mM, phenylmethylsulphonyl fluoride (PMSF), 1-mM dithiothreitol, pH 7.4) per g of tissue on ice in a Polytron. The homogenates were centrifuged at 20 000 *g* for 15 min. The precipitates were

**Figure 1** Analysis of TN-C expression in lung cancer and normal lung tissues by Western (**A**) and Northern (**B**) blotting and dot blot hybridization (**C**). Lanes 1–6, cancer tissues. Lane 7, normal lung

**Figure 2** Analysis of TN-C degradation in lung cancer tissues by Western blotting. (**A**) Cases showing little TN-C degradation. (**B**) Cases showing evident TN-C degradation resuspended in buffer A supplemented with 2 M urea (1 ml per g of tissue), stirred for 2 h and recentrifuged. The supernatants were saved as urea extracts.

The urea extracts (100 μg of protein per lane) were subjected to electrophoresis in 6% SDS–polyacrylamide gels according to the method of Laemmli (1970). Immunoblotting was performed using the procedure of Khyse-Andersen (Khyse-Andersen, 1984). The membranes were incubated with blocking solution containing 2% normal rabbit serum for 30 min, then with RCB1 (final concentration 3.3 mg $ml^{-1}$) for 60 min, following the avidin–biotin complex (ABC) method. Immunoreactive proteins were visualized by reaction in a solution containing 0.03% (w/v) cobalt chloride, 0.04% 3,3-diaminobenzidine tetrachloride and 0.036% hydrogen peroxide.

### Northern blotting and dot blot hybridization

The human TN-C cDNA clone HT24C (nucleotides 6209–7295; Siri et al, 1991) was a kind gift from Dr Zardi, National Cancer Research Institute, Genoa, Italy.

Total RNAs were extracted from the materials by the acid guanidium thiocyanate–phenol–chloroform method (Chomczynski and Sacci, 1987), electrophoresed (10 μg per lane) in 0.8% agarose/2.2 M formaldehyde-denaturing gels and transferred onto Hybond-N filters (Amersham, Buckinghamshire, UK). Blotted membranes were then hybridized with the $P^{32}$-labelled TN-C probe. For dot blot hybridization, 5-μg aliquots of each RNA were dotted onto Hybond-N filters and hybridized simultaneously in the same way as for Northern blotting.

### Immunohistochemistry

Tissues were fixed in phosphate-buffered paraformaldehyde with picric acid and frozen in liquid nitrogen for cryostat sectioning. The sections were immunostained for TN-C as described previously (Ilunga and Iriyama, 1995) using the ABC method.

### RESULTS

### Expression of large TN-C isoforms in lung tissues

On immunoblotting, TN-C reactive bands sized 190 kDa were observed in all 30 of the lung cancers; bands sized 250 kDa, occasionally accompanied by a 220-kDa band, were observed in 28 cases (Figure 1A, lanes 1–6). Abundant expression of TN-C mRNA, in comparison with normal lung was demonstrated in 22 of the 30 cases by dot blot hybridization (Figure 1C, lanes 1–5). The remaining eight cases showed no or little TN-C expression (Figure 1C, lane 6). On Northern blotting, a 7.5-kb large-size TN-C mRNA isoform was found in 19 cases and a 5.5-kb small-size

© Cancer Research Campaign 1998

**Table 1** TN-C in lung tissues and its degradation

| Histological type | No. of cases | Degradation of TN-C (%) | |
|---|---|---|---|
| | | + | – |
| | | n (%) | n (%) |
| Neoplastic | | | |
| Squamous cell carcinoma | 11 | 2 (18.2) | 9 (81.8) |
| Adenocarcinoma | 19 | 5 (26.3) | 14 (73.7) |
| Normal | 6 | 0 | 6 (100) |

**Table 2** Relation of TN-C degradation to lymph node metastasis

| Lymph node metastasis | No. of cases | Degradation of TN-C (%) | |
|---|---|---|---|
| | | + | – |
| | | n (%) | n (%) |
| + | 13 | 6 (46.1)[a] | 7 (53.8) |
| – | 17 | 1 (5.8) | 16 (94.1) |

[a]Significant positive relation between metastasis and TN-C degradation ($P = 0.025$).

isoform in 6 of the 22 cases. Normal lungs expressed only the smaller 5.5-kb TN-C (Figure 1B, lane 7). Immunoreactive TN-C was weakly present in normal lungs at 250 and 190 kDa (Figure 1A, lane 7). The appearance of the high band TN-C molecule is presumably caused by limited expression of the large isoform of TN-C mRNA.

### TN-C degradation in lung cancers

Cases were divided into two groups from the migrating patterns of the TN-C molecules on the gels, one showing two or three bands at the same heights as those of the controls (Figure 2A) and the other exhibiting additional intensely labelled bands lower than 190 kDa (Figure 2B). These bands were concluded to be generated by degradation of TN-C. No relationship was found between the histological type and such degradation (Table 1). In tumours demonstrating lymph node metastasis, however, lower bands were observed in 6 of 13 cases (46.1%), a significantly higher incidence than in the group without metastasis (5.8%) ($P = 0.025$, Table 2).

### Tissue distribution of TN-C demonstrated by immunohistochemistry

Examination of 30 neoplastic tissues by TN-C immunohistochemistry revealed intense and diffuse staining of the fibrous stroma surrounding the neoplastic epithelia in all tumours, regardless of the histological and clinical features. Cases whose TN-C molecules were degraded on the gels did not show any remarkable differences from their non-degraded counterparts (Figure 3A and B). In the six normal lung specimens, TN-C staining was positive in the bronchial basement membrane and the vascular walls, and was irregularly scattered on the alveolar walls (Figure 3C).

**Figure 3** Immunohistochemistry of TN-C in lung cancers and normal lung. (**A**) An adenocarcinoma without TN-C degradation. (**B**) An adenocarcinoma with TN-C degradation. (**C**) Normal lung. Bar 100 μm

### DISCUSSION

In the breast (Koukoulis et al, 1991; Borsi et al, 1992), prostate (Ibrahim et al, 1993) and colorectum (Hauptmann et al, 1995), preferential expression of higher-molecular-weight isoforms of TN-C generated by alternative splicing of RNAs has been observed in cancer tissues. In this study, we demonstrated similar results for the majority of lung cancers. Thus, a large 7.5-kb isoform was detected in all 22 cases expressing appreciable levels of TN-C mRNA, while in normal samples only a small 5.5-kb isoform was found. With regard to the activity of the alternatively spliced domain of TN-C, two papers have appeared in the literature reporting an association with down-regulation of focal adhesion of cells (Murphy-Ullrich et al, 1991) and stimulation of corneal cell migration (Kaplony et al, 1991). Other studies using recombinant fragment of human TN-C have revealed that the alternatively

© Cancer Research Campaign 1998

spliced domain can bind to annexin II on human endothelial cells and promote cell migration (Chung and Erickson, 1994; Chung et al, 1996). These findings suggest that the larger TN-C molecules may be involved in detachment of cells from the stroma and in cell movement, thus affecting the malignant behaviour of tumour cells.

On the other hand, the extracellular matrix (ECM) network forms a tough and highly crosslinked structural skeleton, which may protect against penetration by tumour cells in the tissue. In this regard, a crucial step in tumour invasion is degradation of ECM proteins by proteolytic enzymes. Two papers concerned with degradation of TN-C by matrix metalloproteinase (MMP) have been published. One described the susceptibility of TN-C purified from human melanoma cells (mainly the large isoform) to MMPs-1, -3 and -7 (Imai et al, 1994). The other reported the variation in small and large isoforms purified from baby hamster kidney (BHK) cells transfected with human TN-C cDNA (Siri et al, 1995). Recently, TN-C degradation in tissue was reported by Riley et al (1996) on Western blot analysis of TN-C extracted from normal and degenerate tendons. Only the small isoform was found in normal tendons, while the large isoform was also detected with several proteolytic fragments in degenerate tendons. Here, in lung cancers, we demonstrated several smaller bands of TN-C on Western blots. Although we can not rule out the possibility of spliced variants, the absence of any smaller transcripts than 5.5 kb on the Northern blots strongly suggests the conclusion that these smaller bands are indeed degradation products of TN-C. As MMPs are known to be produced by various tumour cells (Stetler-Stevenson et al, 1993), it is likely that they are also expressed by lung cancers. The pattern of fragmentation of TN-C found in the present study thus suggests the presence of various types of MMPs.

We found a higher frequency of lymph node metastasis in many cases with degraded TN-C molecules. This indicates that the amount of degraded fragments of TN-C may be related to the degree of dynamic remodelling of cancer tissues. According to Siri et al (1995), the large isoform is degraded by MMPs-2 and -3, with cleavage occurring inside the alternatively spliced region. Thus, it is extremely important to identify what proteolytic enzymes are active in lung cancers and how they influence the clinicopathological behaviour.

In the present study, strong immunoreactivity was observed in the stroma of all cases of various histological types. In normal lungs, in contrast, weak staining was found in the bronchial basement membrane and vascular walls in line with earlier findings (Soini et al, 1993). Thus, TN-C positivity is not restricted to malignant neoplasms and is not specific for the cancer tissue, as initially proposed (Mackie et al, 1987). It rather appears to be present at interfaces between different types of cell dynamics. Invasion fronts clearly represent such interfaces, where cancer cells confront host tissues. During cancer progression, preferential expression and deposition of large isoform TN-C might bring about changes conducive to cell movement. Deposited TN-C is finally digested into small fragments by a variety of MMPs. As it proved to be impossible at the immunohistochemistry level to evaluate the extent of degradation of TN-C molecules, biochemical analyses may be more useful for further clinicopathological assessment of the role that this process plays in malignancy.

### ABBREVIATIONS

TN-C, tenascin-C; ECM, extracellular matrix; SCC, squamous cell carcinoma; MMP, matrix metalloproteinase

### ACKNOWLEDGEMENTS

We thank Drs Minoru Kusagawa, Ryuichi Yatani and Tsutomu Okinaka for their valuable suggestions. We are grateful to Dr Malcolm A Moore for his editorial assistance. This work was supported in part by Grants-in-Aid for General Scientific Research from the Ministry of Education, Science, Sports and Culture, Japan, and the Mie Medical Research Foundation.

### REFERENCES

Anbazhagen R, Sakakura T and Gusterson BA (1990) The distribution of immunoreactive tenascin in the epithelial–mesenchymal junctional areas of benign and malignant squamous epithelia. *Virchows Arch B Cell Pathol* **59:** 59–63

Borsi L, Carnemolla B, Nicolo G, Spina B, Tanara G and Zardi L (1992) Expression of different tenascin isoforms in normal, hyperplastic and neoplastic human breast tissues. *Int J Cancer* **52:** 688–692

Bourdon MA, Wikstrand CJ, Furthmayr H, Matthews TJ and Bigner DD (1983) Human glioma mesenchymal extracellular matrix antigen defined by monoclonal antibody. *Cancer Res* **43:** 2796–2805

Chiquet-Ehrismann R, Mackie EJ, Pearson AC and Sakakura T (1986) Tenascin: an extracellular matrix protein involved in tissue interactions during fetal development and oncogenesis. *Cell* **47:** 131–139

Chomczynski P and Sacci N (1987) Single-step method of RNA isolation by acid guanidium thiocyanate–phenol–chloroform extraction. *Anal Biochem* **162:** 156–159

Chung CY and Erickson HP (1994) Cell surface annexin II is a high affinity receptor for the alternatively spliced segment of tenascin-C. *J Cell Biol* **126:** 539–548

Chung CY, Murphy-Ullrich JE and Erickson HP (1996) Mitogenesis, cell migration, and loss of focal adhesions induced by tenascin-C interacting with cell surface receptor, annexin II. *Mol Biol Cell* **7:** 883–892

Erickson HP and Bourdon MA (1989) Tenascin: an extracellular matrix protein prominent in specialized embryonic tissues and tumors. *Annu Rev Cell Biol* **5:** 71–92

Hauptmann S, Zardi L, Siri A, Carnemolla B, Borsi L, Castellucci M, Klosterhalfen B, Hartung P, Weis J, Stocker G, Haubeck H-D and Kirkpatrick CJ (1995) Extracellular matrix proteins in colorectal carcinomas. Expression of tenascin and fibronectin isoforms. *Lab Invest* **73:** 172–182

Ibrahim SN, Lightner VA, Ventimiglia JB, Ibrahim JK, Walther PJ, Bigner DD and Humphrey PA (1993) Tenascin expression in prostatic hyperplasia, intraepithelial neoplasia, and carcinoma. *Hum Pathol* **24:** 982–989

Ilunga K and Iriyama K (1995) Expression of tenascin in gastric carcinoma. *Br J Surg* **82:** 948–951

Imai K, Kusakabe M, Sakakura T, Nakanishi I and Okada Y (1994) Susceptibility of tenascin to degradation by matrix metalloproteinases and serine proteinases. *FEBS Lett* **352:** 216–218

Jones FS and Copertino DW (1996) The molecular biology of tenascin: structure, splice variants, and regulation of gene expression. In *Tenascin and Counteradhesive Molecules of the Extracellular Matrix*, Crossin KL. (ed.), pp. 1–22, Harwood Academic: The Netherlands

Kaplony A, Zimmermann DR, Fischer RW, Imhof BA, Odermatt BF, Winterhalter KH and Vaughan L (1991) Tenascin Mr 220,000 isoform expression correlates with corneal cell migration. *Development* **112:** 605–614

Khyse-Andersen J (1984) Electroblotting of multiple gels: a simple apparatus without buffer tank for rapid transfer of proteins from polyacrylamide of nitrocellulose. *J Biochem Biophys Meth* **10:** 203–209

Koukoulis GK, Gould VE, Bhattacharyya A, Gould JK, Howeedy AA and Virtanen I (1991) Tenascin in normal, reactive, hyperplastic, and neoplastic tissues: biologic and pathologic implications. *Hum Pathol* **22:** 636–643

Laemmli UK (1970) Cleavage of structural proteins during assembly of the head of the bacteriophage. *Nature* **227:** 680–685

Mackie EJ, Chiquet-Ehrismann R, Pearson AC, Inaguma Y, Taya K, Kawarada Y and Sakakura T (1987) Tenascin is a stromal marker for epithelial malignancy in mammary gland. *Proc Natl Acad Sci USA* **84:** 4621–4625

Murphy-Ullrich J, Lightner VA, Aukhil I, Erickson HP and Hook M (1991) Focal adhesion integrity is downregulated by the alternatively spliced domain of human tenascin. *J Cell Biol* **115:** 1127–1136

Oike Y, Hiraiwa H, Kawakatsu H, Nishikai M, Okinaka T, Suzuki T, Okada A, Yatani R and Sakakura T (1990) Isolation and characterization of human fibroblast tenascin. An extracelluar matrix glycoprotein of interest for developmental studies. *Int J Dev Biol* **34:** 309–317

© Cancer Research Campaign 1998

Oyama F, Hirohashi S, Shimosato Y, Titani K and Sekiguchi K (1991) Qualitative and quantitative changes of human tenascin expression in transformed lung fibroblast and lung tumor tissues: comparison with fibronectin. *Cancer Res* **51:** 4876–4881

Riley GP, Harrall RL, Cawston TE, Hazleman BL and Mackie EJ (1996) Tenascin-C and human tendon degeneration. *Am J Pathol* **149:** 933–943

Sakakura T (1995) role of tenascin in cancer development. In *Tumor Matrix Biology*, Adany R. (ed.), pp. 101–129. CRC Press: Boca Raton

Sakakura T, Ishihara A and Yatani R (1991) Tenascin in mammary gland development: from embryogenesis to carcinogenesis. In *Regulatory Mechanisms in Breast Cancer*, Lippman M and Dickson R. (eds), pp. 383–400. Kluwer Academic: Boston

Siri A, Carnemolla B, Saginati M, Leprini A, Casari G, Baralle F and Zardi L (1991) Human tenascin: primary structure, pre-mRNA splicing patterns and localization of the epitopes recognized by two monoclonal antibodies. *Nucleic Acids Res* **19:** 525–531

Siri A, Knauper V, Veirana N, Caocci F, Murphy G and Zardi L (1995) Different susceptibility of small and large human tenascin-C isoforms to degradation by matrix metalloproteinases. *J Biol Chem* **270:** 8650–8654

Soini Y, Paakko P, Nuorva K, Kamel D., Linnala A, Virtanen I and Lehto V-P (1993) Tenascin immunoreactivity in lung tumors. *Am J Clin Pathol* **100:** 145–150

Stetler-Stevenson WG, Aznzvoorian S and Liotta LA (1993) Tumor cell interactions with the extracellular matrix during invasion and metastasis. *Annu Rev Cell Biol* **9:** 541–573

Van Eyken P, Sciot R and Desmet VJ (1990) Expression of the novel extracellular matrix component tenascin in normal and diseased human liver: an immunohistochemical study. *J Hepatol* **11:** 43–52

Vollmer G, Siegal GP, Chiquet-Ehrismann R, Lightre VA, Arnholdt H and Knuppen R (1990) Tenascin expression in the human endometrium and in endometrial adenocarcinoma. *Lab Invest* **62:** 725–730

*© Cancer Research Campaign 1998*